# SARS-CoV-2 and neurotropism: evidence, gaps and reflections

**DOI:** 10.1099/jmm.0.002016

**Published:** 2025-06-16

**Authors:** Thaísa Regina Rocha Lopes, Bibiana Santana Sitton, Micheli Mainardi Pillat, Carlos Fernando Mello, Rudi Weiblen, Eduardo Furtado Flores, José Valter Joaquim Silva Júnior

**Affiliations:** 1 Setor de Virologia, Departamento de Medicina Veterinária Preventiva, Universidade Federal de Santa Maria, Santa Maria, Rio Grande do Sul, Brazil; 2 Programa de Pós-graduação em Medicina Veterinária, Universidade Federal de Santa Maria, Santa Maria, Rio Grande do Sul, Brazil; 3 Laboratório NB3 de Neuroimunologia, Universidade Federal de Santa Maria, Santa Maria, Rio Grande do Sul, Brazil; 4 Programa de Pós-graduação em Farmacologia, Universidade Federal de Santa Maria, Santa Maria, Rio Grande do Sul, Brazil; 5 Departamento de Microbiologia e Parasitologia, Universidade Federal de Santa Maria, Santa Maria, Rio Grande do Sul, Brazil; 6 Setor de Virologia, Instituto Keizo Asami, Universidade Federal de Pernambuco, Recife, Pernambuco, Brazil

**Keywords:** coronavirus, tropism, coronavirus disease 2019 (COVID-19)

## Abstract

Coronavirus disease 2019 (COVID-19) patients may present with a wide clinical spectrum, including extrapulmonary involvement, such as neurological damage. Although the pathogenesis of neurological COVID-19 still remains unclear, some studies have discussed the potential association between tissue injury and severe acute respiratory syndrome-related coronavirus 2 (SARS-CoV-2) infection in the central nervous system (CNS) and/or immune imbalance. These two mechanisms are non-mutually exclusive; however, exacerbated inflammatory-response-induced neurological damage appears to be more aligned with COVID-19 pathogenesis, whereas SARS-CoV-2 infection/replication in the CNS remains widely discussed. Herein, we dissect this last issue, highlighting some evidence on SARS-CoV-2 neuroinvasion, as well as discussing gaps that should be addressed for a better understanding of its potential neurotropism, specifically in the CNS. Finally, we propose some deeper reflections on the SARS-CoV-2 neurotropic potential.

**Dear Editor,**

Severe acute respiratory syndrome-related coronavirus 2 (SARS-CoV-2) infections result primarily in respiratory disease; however, atypical clinical signs and extrapulmonary damage, such as neurological involvement, have also been reported in coronavirus disease 2019 (COVID-19) patients [1]. Neurological injury may be related to SARS-CoV-2 infection/replication in the central nervous system (CNS) (direct mechanism) and/or to an imbalance in the immune response (indirect mechanism), particularly in severe cases [2]. Although these mechanisms are non-mutually exclusive, exacerbated inflammatory-response-induced neurological damage appears to be more closely aligned with COVID-19 pathogenesis, whereas SARS-CoV-2 infection/replication in the CNS remains widely discussed [2]. To contribute to this issue, we highlight some consolidated findings on SARS-CoV-2 neuroinvasion, as well as discuss some gaps that should be addressed for a better understanding of its neurotropic potential, specifically in the CNS.

First, SARS-CoV-2 or at least some of its antigens, such as spike (S) protein, nucleoprotein (N) and viral RNA (vRNA), may have access/invade the CNS; a conclusion supported by several study models, including two-dimensional (2D) cell culture, organoids, non-human primates (NHPs) and post-mortem analyses (humans) [3,13]. The expression of angiotensin-converting enzyme 2 (ACE2) and other possible receptors for SARS-CoV-2 (e.g. neuropilin-1, CD147 and dipeptidyl peptidase-4/CD26) in the CNS also supports its potential neuroinvasiveness [14,16]. Furthermore, possible routes for SARS-CoV-2 entry into the CNS, such as through the blood-cerebrospinal fluid barrier (BCSFB) and/or olfactory nerves, have also been discussed [4,5, 9, 13, 17]. The blood-brain barrier (BBB), whose integrity could be overcome/compromised by direct viral or inflammation-related factors, may also be a potential pathway for SARS-CoV-2 neuroinvasion [16,18].

Despite the contribution of these studies, the question that still needs to be further explored is whether, once invading the CNS, SARS-CoV-2 would be capable of sustained productive infection, which would prove its neurotropism. *In vitro* studies performed with 2D cell culture or brain organoids have led to different findings on this issue, demonstrating or suggesting abortive or productive SARS-CoV-2 infection [4,6, 10, 11, 19, 20]. These divergent results may be influenced by differences in experimental design, cell line, SARS-CoV-2 isolate/strain or multiplicity of infection used in some studies. Nevertheless, this is a noteworthy point, especially since cellular models, in general, should be more easily infected when compared with an *in vivo* system, where several obstacles, such as biological barriers and immunological components, limit access and/or replication in the CNS. Overall, this scenario would point against the SARS-CoV-2 neurotropic potential.

Analyses performed with animal models also suggest something similar to what is described above. Experiments with NHPs (rhesus monkeys), for example, describe detection of vRNA, double-stranded RNA (dsRNA) and/or viral protein (S/ N protein) in the CNS [3,5], without mentioning viral isolation, which would be indicative of viable virus and potential neurotropism, or detection of subgenomic viral RNA (sgRNA), which could be used as a marker of viral replication [21,22]. The identification of mutations in vRNA recovered from CNS versus respiratory tissue vRNA, which could suggest independent viral replication at different sites [22], has also not been mentioned in these experiments. Furthermore, we believe that the detection of SARS-CoV-2 non-structural proteins (NSPs) would also contribute to the investigation of its neurotropic potential. Since NSPs are not usually found in viral particles [23], their detection in CNS cells would be more suggestive of viral replication than the finding of structural proteins, such as S and N proteins, which could also be molecular residues remaining from viral entry. The detection of NSPs in SARS-CoV-2-infected cells could be performed by simple assays, such as immunofluorescence or immunohistochemistry [24, 25].

Importantly, although some findings may vary depending on the NHP used, as well as the experimental design adopted [26], the overwhelming majority of COVID-19 post-mortem analyses (humans) also describe only the detection of viral protein (S and/or N protein) and/or vRNA in the CNS (as reviewed by Mukerji and Solomon [8]), without viral isolation, detection of sgRNA/NSPs or identification of different sequences for vRNA from the CNS. In fact, these *in vitro* (2D cell culture and brain organoid) and *in vivo* (NHP and humans) studies are important to demonstrate the potential neuroinvasion of SARS-CoV-2 and/or its antigens, but they still do not answer questions regarding its neurotropism. It is a fact that some studies have demonstrated replication (or marker of replication) of SARS-CoV-2, as well as SARS-CoV, in the CNS of transgenic mice with increased expression of ACE2, the receptor for both SARS-CoV and SARS-CoV-2 [27,29]. Although this animal model may be useful for investigating some pathogenic mechanisms of COVID-19, altered ACE2 receptor expression may bias inferences about SARS-CoV-2 tropism.

On the other hand, some post-mortem analyses of COVID-19 patients have supported the neurotropic potential of SARS-CoV-2 [22,30]. Stein *et al*. [22] detected sgRNA in the CNS and performed viral isolation from the thalamus and hypothalamus, in addition to identifying additional mutations in vRNA recovered from the CNS. These findings raise important questions for discussion. For instance, isolation of SARS-CoV-2 from the thalamus and hypothalamus was only possible in Vero E6-TMPRSS2-T2A-ACE2 [22], a cell highly susceptible to SARS-CoV-2 infection, but not in Vero E6, which is widely used for SARS-CoV-2 isolation [31]. Interestingly, these samples had a similar Cq/copy number to those from respiratory tissue that were successfully isolated in Vero E6 [22]. Considering this scenario, it may be useful to explore the hypothesis that mutations in brain-derived SARS-CoV-2 could influence its replication in Vero E6, which would help explain the observed results, as well as contribute to future investigations on viral infection dynamics. This suggestion/hypothesis is in line with the previous observation that different SARS-CoV-2 variants may present different replication kinetics in Vero E6 [32].

Furthermore, Stein *et al*. [22] found no histological alterations indicative of a robust inflammatory process in the CNS, even in the face of the considerable viral load found in the tissue. Importantly, some potential biases, such as residual blood in the tissues or cross-contamination of the lungs during sample collection, were also ruled out by Stein *et al*. [22]. Overall, these findings add more layers to the investigation of the SARS-CoV-2-CNS relationship, pointing in the opposite direction to that previously discussed and supporting the SARS-CoV-2 neurotropism.

Finally, despite the importance of all studies for understanding the SARS-CoV-2-CNS interplay, we believe that there are still not enough elements to confirm or discard its neurotropic potential. Moreover, we also believe it is appropriate to highlight at least three points for reflection: (i) what would explain the fact that even 5 years after the first description of SARS-CoV-2, and with unprecedented efforts to investigate it, there is no concrete answer about its potential neurotropism?; (ii) when a virus is truly neurotropic, even if it is a new or emerging agent, shouldn't its ability to replicate in the CNS be observed without much difficulty and described more consistently, in addition to being more easily reproduced under laboratory conditions, as observed for the Zika virus?; and (iii) finally, regardless of past or future findings, it is important to remember the maxim ‘truth is the daughter of time’ and remain alert for the emergence of SARS-CoV-2 strains with varied tropisms.

## Abbreviations

ACE2: angiotensin-converting enzyme 2
CNS: central nervous system
COVID-19: coronavirus disease 2019
N: nucleoprotein
NHPs: non-human primates
NSPs: non-structural proteins
S: spike
SARS-CoV-2: severe acute respiratory syndrome-related coronavirus 2
sgRNA: subgenomic viral RNA
vRNA: viral RNA
